# Mesenchymal stem cell-derived exosomes ameliorate cardiomyocyte apoptosis in hypoxic conditions through microRNA144 by targeting the PTEN/AKT pathway

**DOI:** 10.1186/s13287-020-1563-8

**Published:** 2020-01-23

**Authors:** Zhuzhi Wen, Zun Mai, Xiaolin Zhu, Tao Wu, Yangxin Chen, Dengfeng Geng, Jingfeng Wang

**Affiliations:** 1 Department of Cardiology, Sun Yat-sen Memorial Hospital, Sun Yat-sen University, 107 Yangjiang West Road, Guangzhou, 510120 China; 2 Guandong Province Key Laboratory of Arrhythmia and Electrophysiology, Guangzhou, China; 3 RNA Biomedical Institute, Sun Yat-Sen Memorial Hospital, Sun Yat-Sen University, Guangzhou, China; 4 Breast Tumor Center, Sun Yat-Sen Memorial Hospital, Sun Yat-Sen University, Guangzhou, China

**Keywords:** Mesenchymal stem cells, Exosomes, microRNA-144, PTEN/AKT pathway, Cell apoptosis

## Abstract

**Background:**

A growing body of evidence suggests that stem cell-derived exosomal microRNAs (miRNAs) could be a promising cardioprotective therapy in the context of hypoxic conditions. The present study aims to explore how miRNA-144 (miR-144), a miRNA contained in bone marrow mesenchymal stem cell (MSC)-derived exosomes, exerts a cardioprotective effect on cardiomyocyte apoptosis in the context of hypoxic conditions and identify the underlying mechanisms.

**Methods:**

MSCs were cultured using the whole bone marrow adherent method. MSC-derived exosomes were isolated using the total exosome isolation reagent and confirmed by nanoparticle trafficking analysis as well as western blotting using TSG101 and CD63 as markers. The hypoxic growth conditions for the H9C2 cells were established using the AnaeroPack method. Treatment conditions tested included H9C2 cells pre-incubated with exosomes, transfected with miR-144 mimics or inhibitor, or treated with the PTEN inhibitor SF1670, all under hypoxic growth conditions. Cell apoptosis was determined by flow cytometry using 7-ADD and Annexin V together. The expression levels of the miRNAs were detected by real-time PCR, and the expression levels of AKT/p-AKT, Bcl-2, caspase-3, HIF-1α, PTEN, and Rac-1 were measured by both real-time PCR and western blotting.

**Results:**

Exosomes were readily internalized by H9C2 cells after co-incubation for 12 h. Exosome-mediated protection of H9C2 cells from apoptosis was accompanied by increasing levels of p-AKT. MiR-144 was found to be highly enriched in MSC-derived exosomes. Transfection of cells with a miR-144 inhibitor weakened exosome-mediated protection from apoptosis. Furthermore, treatment of cells grown in hypoxic conditions with miR-144 mimics resulted in decreased PTEN expression, increased p-AKT expression, and prevented H9C2 cell apoptosis, whereas treatment with a miR-144 inhibitor resulted in increased PTEN expression, decreased p-AKT expression, and enhanced H9C2 cell apoptosis in hypoxic conditions. We also validated that PTEN was a target of miR-144 by using luciferase reporter assay. Additionally, cells treated with SF1670, a PTEN-specific inhibitor, resulted in increased p-AKT expression and decreased H9C2 cell apoptosis.

**Conclusions:**

These findings demonstrate that MSC-derived exosomes inhibit cell apoptotic injury in hypoxic conditions by delivering miR-144 to cells, where it targets the PTEN/AKT pathway. MSC-derived exosomes could be a promising therapeutic vehicle to facilitate delivery of miRNA therapies to ameliorate ischemic conditions.

**Electronic supplementary material:**

The online version of this article (10.1186/s13287-020-1563-8) contains supplementary material, which is available to authorized users.

## Background

Ischemic heart disease resulting in chronic heart failure is a leading cause of mortality and morbidity worldwide. Since Makino et al. induced cardiomyocytes (CMCs) by treating bone marrow mesenchymal stem cells (MSCs) with 5-azacytidine in vitro in 1999 [1], MSCs have quickly become a widely used experimental model and have been used in a number of clinical trials. MSC-based therapy is emerging as a novel approach to repair ischemic myocardial damage. Paracrine effects likely account for the majority of mechanisms by which MSCs participate in ischemic cardiac repair [2–4].

Recent studies have shown that MSC-based treatment of ischemia-induced cardiac damage can be mediated through the secretion of exosomes [5–9]. MSC-secreted exosomes have been reported to exert an anti-apoptotic effect on CMCs both in vivo and in vitro [5–7, 10]. Exosomes exert their therapeutic effect by transferring lipids, proteins, and a variety of RNAs to recipient cardiac cells. RNAs transferred in MSC exosomes are of great interest, given that there are a variety of highly expressed microRNAs (miRNA, miR) contained in MSC-derived exosomes that may act in a paracrine manner to mediate cardiac repair [11–13].

Several studies have shown that in the context of hypoxic conditions, ischemia decreases miR-144 levels, while increasing miR-144 levels in the ischemic heart resulted in cardioprotective benefits, with improved functional recovery and remodeling of the heart [14–16]. MiR-144 is influential in the proliferation, differentiation, growth, and death processes of cells, and the impact of miR-144 on CMC apoptosis in hypoxic conditions has been elucidated in these studies. MiR-144 has demonstrated the potential to be utilized as a novel therapeutic approach for treating ischemia-induced myocardial damage [17]. MSC-derived exosomes can protect the heart from ischemia by increasing the level of miR-21a-5p in recipient cardiac cells, thereby downregulating expression of the pro-apoptotic gene phosphatase and tensin homolog deleted on chromosome 10 (PTEN) in the myocardium [7]. PTEN has been confirmed as a direct target of miR-144 [18–20], and miR-144 inhibited cell apoptosis through inhibition of PTEN and subsequent activation of the phosphatidylinositol 3-kinase (PI3K)/protein kinase B (AKT) signaling pathway [19].

However, a relationship between miR-144 regulation of PTEN/AKT signaling and the anti-apoptotic effect of MSC-derived exosomes on CMCs grown in hypoxic conditions has yet to be established. Therefore, the present study aimed to characterize whether miR-144 delivered to CMCs in MSC-derived exosomes exerted anti-apoptotic effects on CMCs in hypoxic growth conditions, and to explore whether the PTEN/AKT signaling pathway or other putative miR-144 targets play a role in regulating CMC apoptosis in hypoxic conditions.

## Methods

### Isolation and culture of bone marrow MSCs

Bone marrow MSCs were isolated from 1-month-old Sprague–Dawley rats as described previously [21]. Briefly, total bone marrow cells were flushed from femurs and tibias using cell culture media under sterile conditions. The cells were then seeded into 25-cm^2^ culture flasks (Corning) containing complete culture media (GIBCO) supplemented with 10% fetal bovine serum (FBS) (GIBCO), penicillin (100 IU/ml), and streptomycin (100 μg/ml) (GIBCO). Cells were cultured at 37 °C in an incubator with a humidified atmosphere containing 5% carbon dioxide (Thermo). The media were replaced every 48 h to remove nonadherent cells. When the adherent cells reached 90% confluency, they were harvested with 0.25% trypsin-ethylenediamine tetraacetic acid (GIBCO) and passaged. MSCs were characterized at passage 3 via fluorescence-activated cell sorting, as reported previously [22] and cells from the same batch were used for the extraction of exosomes (described below).

### Harvest and identification of MSC exosomes

MSC-derived exosomes were harvested from passage 3 MSCs grown to 80% confluency in 10-cm plates (Corning) using the total exosome isolation reagent (Life Technology). The complete media were aspirated and the cells were washed three times with phosphate-buffered saline (PBS) (GIBCO). Serum-free media were then added to the cells, and after 36 h, the media was collected and centrifuged at 3000*g* at 4 °C for 30 min, then transferred to new tubes and centrifuged at 16000*g* at 4 °C for 20 min. The media were filtered using a 0.22-μm filter (Millipore), before being carefully transferred to an ultrafiltration device with 30-kDa cutoff (Millipore) and centrifuged at 6000*g* at 4 °C for 15 min. The concentrate was obtained after the removal of cellular debris. This procedure was repeated to collect enough concentrate for experiments. The concentrate was transferred to a new tube, and the total exosome isolation reagent was added at a ratio of 1: 2 to the concentrate. The tubes were then vortexed to make a homogenous solution. The homogenous solution was incubated overnight at 4 °C and then centrifuged at 4 °C at 10,000*g* for 1 h. The supernatant was removed, and the pellets containing exosomes were resuspended with 500 μl PBS and then centrifuged at 4 °C at 10,000*g* for 5 min. After decanting and aspirating residual liquid, exosomes were obtained and stored at − 80 °C until use.

A 500 μl exosome solution in PBS was used for bovine serum albumin (BSA) protein quantitation, western blotting, nanoparticle trafficking analysis (NTA), and cell treatment. NTA was used to identify exosomes. Analysis of the absolute size distribution of exosomes was performed using a NanoSight NS300 (Malvern). Briefly, approximately 2 μl exosome solution was diluted in 1 ml of PBS and vortexed to mix. The exosomes were completely resuspended using an ultrasonicator, and then the exosome suspension was extracted and injected into the NanoSight NS300 detector. Control media and PBS alone were used as controls. Each sample was analyzed in triplicate. The presence of exosomes was confirmed by western blotting using the exosomal markers TSG101 and CD63.

### H9C2 cell culture and treatment

H9C2 CMCs of rat cardiac origin were obtained from Guangzhou Cellcook Biotech Co., Ltd., China. Cells were cultured with high glucose Dulbecco’s modified Eagle’s medium (GIBCO) supplemented with 10% FBS (GIBCO) in a CO_2_ incubator kept at 37 °C with a humid atmosphere of 95% air, 5% CO_2_ (Thermo). When cells became 80% confluent, they were harvested and passaged using trypsin-ethylenediamine tetraacetic acid (GIBCO). The hypoxic cell growth conditions were established using the AnaeroPack method as previously described in other studies [23–25]. Briefly, H9C2 cells cultured in six-well culture plates (Corning) were placed into a sealed airtight container. An AnaeroPack™ MicroAero (Mitsubishi Gas Chemical, Tokyo, Japan) was placed inside the container to generate a hypoxic atmosphere by absorbing oxygen and producing carbon dioxide. Cells were incubated in the hypoxic container for 48 h at 37 °C in a CO_2_ incubator (hypoxia treatment group). Increasing concentrations of exosomes were added to the culture media (media lacking exosomes, 1, 5, 25, and 50 μg/ml); H9C2 cells were pre-incubated in the media containing MSC-derived exosomes in normal growth conditions for 24 h prior to exposure to hypoxic conditions. Cells that were grown in normoxia in media containing 5 μg/ml of exosomes for 24 h and then switched to hypoxic conditions for 48 h were defined as the hypoxia+exosome group. H9C2 cells transfected with miR-144 mimics or miR-144 inhibitors and their corresponding scrambled controls were also incubated in hypoxic conditions (hypoxia + miR-144 mimics/inhibitor group, hypoxia + mimics/inhibitor control group, respectively). Transfected cells were also treated with exosomes for 24 h prior to incubation in hypoxic conditions (hypoxia + exosomes + miR-144 mimics or inhibitor). SF1670, a PTEN-specific inhibitor, was used in combination with either added exosomes or transfection of miR-144 mimic to treat cells incubated in hypoxic conditions (hypoxia + exosome +SF1670 group, hypoxia + miR-144 mimics +SF1670 group). Cells incubated in a humid atmosphere of 95% air and 5% CO_2_ at 37 °C were defined as the normoxia group.

### Internalization of MSC-derived exosomes into H9C2 cells

The purified MSC-derived exosomes were labeled using the Exo-GLOW™ Exosome Labeling Kit (SBI) according to the manufacturer’s protocol. Briefly, the exosome pellet was resuspended in 450 μl PBS. Fifty microliters 10 × Exo-Green was added to the exosome resuspension in a 1.5-ml Eppendorf tube, making a 500-μl esosome suspension. The exosome solution was mixed by inversion and then incubated at 37 °C for 10 min. A total of 100 μl of the ExoQuick-TC reagent was added to the labeled exosome suspension and mixed by inverting six times. The labeled exosome suspension was kept at 4 °C for 30 min and then spun down in a centrifuge at 14,000 rpm for 3 min. After removing the supernatant, the labeled exosome pellet was resuspended in 100 μl PBS. A total of 100 μl of labeled exosomes was added to approximately 1 × 10^5^ cells per well in a six-well culture plate (Corning) and incubated for 12 h, and visualized using fluorescence microscopy. The cells that had been incubated with labeled exosomes were further stained with 7-AAD (BD Biosciences) and phalloidin (BD Biosciences), and cell images were acquired with a confocal laser scanning microscope. The cells that had taken up labeled exosomes were quantified from three independent visual fields per sample, and the mean number of labeled exosomes from three experiments was used for statistical analysis.

### Transfection of H9C2 cells

For testing miR-144 gain-and-loss-of-function, H9C2 cells were transfected with miR-144 mimics or a miR-144 inhibitor, as well as corresponding scrambled controls. MiR-144 mimics and the miR-144 inhibitor, as well as their corresponding scrambled controls (GenePharma, Shanghai, China) were transfected into H9C2 cells using Lipofectamine™ 2000 (GIBCO) based on the manufacturer’s instructions. Cells were incubated in 24-well plates (Corning) and subjected to transfection at 80% confluency. Opti-MEM reduced serum medium (GIBCO) was used for transfection, and the media were changed 4 h after transfection. Real-time polymerase chain reaction (PCR) was performed to confirm the efficiency of mimic and inhibitor transfection, respectively.

Rno-miR-144 mimics:

5′-UACAGUAUAGAUGAUGUACU-3′, 

5′-UACAUCAUCUAUACUGUAUU-3′.

Mimic control:

5′-UUCUCCGAACGUGUCACGUTT-3′, 

5′-ACGUGACACGUUCGGAGAATT-3′.

Rno-miR-144 inhibitor: 5′-AGUACAUCAUCUAUACUGUA-3′.

Inhibitor control: 5′-CAGUACUUUUGUGUAGUACAA-3′.

### Measurement of H9C2 cell apoptosis

Apoptosis of H9C2 cells was determined using flow cytometry following experimental treatment. Cells were treated with trypsin without ethylenediamine tetraacetic acid (GIBCO), collected, and washed twice with ice-cold PBS. Cells were then resuspended in 1× binding buffer at a concentration of 1 × 10^6^ cells/ml. Five microliters Annexin V (BD Biosciences) and 5 μl 7-AAD (BD Biosciences) were added to 100 μl of the cell suspension in a flow tube, mixed well, and incubated for 15 min at room temperature in the dark. Four hundred microliters binding buffer was then added, the tubes were mixed well, and the samples were then analyzed for apoptosis using flow cytometry (BD Biosciences). Each experiment was repeated three times for statistical analysis.

### Real-time PCR

Total RNA was isolated from cells or MSC-derived exosomes using Trizol (GIBCO Invitrogen) and the isolated RNA was quantified by spectrophotometry. Equal amounts of RNA were used for cDNA synthesis using Moloney murine leukemia virus reverse transcriptase (Promega) and the appropriate primers (Additional file 1: Table S1). Real-time PCR was performed on an ABI PRISM® 7500 Sequence Detection System using ChamQ SYBR qPCR Master Mix (Vazyme) as described previously [21, 26]. The relative expression levels of the target genes of interest were calculated using the 2^−ΔΔCt^ method. Fold changes in target gene expressions were calculated after normalizing to U6 or GAPDH expression levels using the 2^−ΔΔCt^ method. Primers for U6 and GAPDH were included in each miRNA and mRNA reaction, as an internal control, respectively. Three repeated experiments were performed for statistical analysis.

Rno-miR-144-3p-RT: GTCGTATCCAGTGCAGGGTCCGAGGTATTCGCACTGGATACGACAGTACA.

Rno-miR-144-3p-F: GCGGGTACAGTATAGATGATG.

Universe-R: GTGCAGGGTCCGAGGT.

### Western blotting

Western blotting was performed as described previously [26]. Briefly, total proteins were extracted from H9C2 cells or MSC-derived exosomes using modified RIPA buffer. Equivalent amounts of proteins were separated on a 10% polyacrylamide gel, transferred to polyvinylidene difluoride membranes, blocked with 5% BSA, and then incubated with anti-TSG101 (Abcam, UK, 1:5000), anti-CD63 (Abcam, UK, 1:1000), anti-PTEN (CST, USA, 1:1000), anti-Ras-related C3 botulinum toxin substrate 1 (Rac-1) (Abcam, UK, 1;200), anti-phosphorylated AKT (*p*-AKT) (CST, USA, 1:1000), anti-AKT (CST, USA, 1: 1000), anti-B-cell lymphoma-2 (Bcl-2) (Abcam, UK, 1:1000), anti-caspase-3 (CST, USA, 1:1000), anti-hypoxia inducible factor-1α (HIF-1α) (Abcam, UK, 1:500), and anti-GAPDH antibodies (Transgen Biotech, 1:3000) at 4 °C overnight. The membranes were washed, incubated with horseradish peroxidase-conjugated secondary antibodies, and visualized using the ECL chemiluminescence system. Densitometry analysis was performed using the Bio-Rad image detection system and Quantity One software (Bio-Rad, CA, USA). After quantifying the band intensities by densitometry, relative steady-state protein levels were calculated after normalizing to GAPDH. Each experiment was performed three times for statistical analysis.

### Transfection of cells with plasmids and luciferase reporter assay

The PTEN wild-type and mutant 3′ untranslated region (UTR) DNA sequences were amplified by PCR techniques using primers as follows: wild-type forward, 5′-CTAGTTGTTTAAACGAGCTCCCATGTTTAGTTTTAGAAAA-3′, and reverse, 5′ -TTGCATGCCTGCAGGTCGACATATATATTCTATATGAAAA-3′; mutant forward, 5′-TTACGCAAAAATATGACATAATGTGTCCTGCATGCAGGCG-3′, and reverse, 5-ATTATGTCATATTTTTGCGTAACGAGGCACTTGTGGCAAC-3′. The wild-type or mutant PTEN 3′UTR luciferase vectors were generated by inserting the amplified DNA sequences into the pmirGLO vector (General Biosystems, China).

H9C2 cells grown to 80% confluency in six-well plates were transfected with pmirGLO vector and miR-144 mimics or scrambled control using the Lipofectamine® 2000 transfection reagent (Life Technologies). Briefly, the media were aspirated and cells were washed twice with PBS, then 1.5 ml basic media were added to the cells in each well. Two micrograms plasmids containing wild-type or mutant PTEN 3′UTR vector, along with miR-144 mimics or scrambled control, were taken and dissolved in 250 μl opti-MEM, mixed well and placed stably. Five microliters Lipofectamine® 2000 transfection reagent was taken to dissolve in 250 μl opti-MEM, mixed gently, and placed stably at room temperature for 5 min. These opti-MEM solutions were mixed well and then placed stably at room temperature for 5 min. The mixed solution containing the wild-type or mutant PTEN 3′UTR plasmids and miR-144 mimics or scrambled control were dripped into the wells and mixed well, and then the cells were incubated at 37 °C in a CO_2_ incubator. After 4 h, the transfected media were aspirated, and 2 ml complete media were added to the cells to continue culture.

For the luciferase reporter assay, the cells were harvested and lysed 48 h after the transfection, and the luciferase assays were performed using the TransDetect Double-Luciferase Reporter Assay Kit (TransGen, Beijing, China) according to the manufacturer’s protocol. The luciferase activity was measured using the ECL chemiluminescence system, and the relative luciferase activity normalized to the corresponding Renilla luciferase activity was obtained. Each experiment was repeated three times for statistical analysis.

### Statistical analysis

All quantitative data are presented as mean ± SD. Data were compared using an independent samples *t*-test. Two-tailed *P* values < 0.05 were considered statistically significant, and adjusted *P* values were used among the subgroup comparison analyses. All statistical analyses were performed using the software package SPSS 22.0 (IBM, USA) for Windows.

## Results

### Exosome isolation, identification, and internalization into cells

**Fig. 1:**
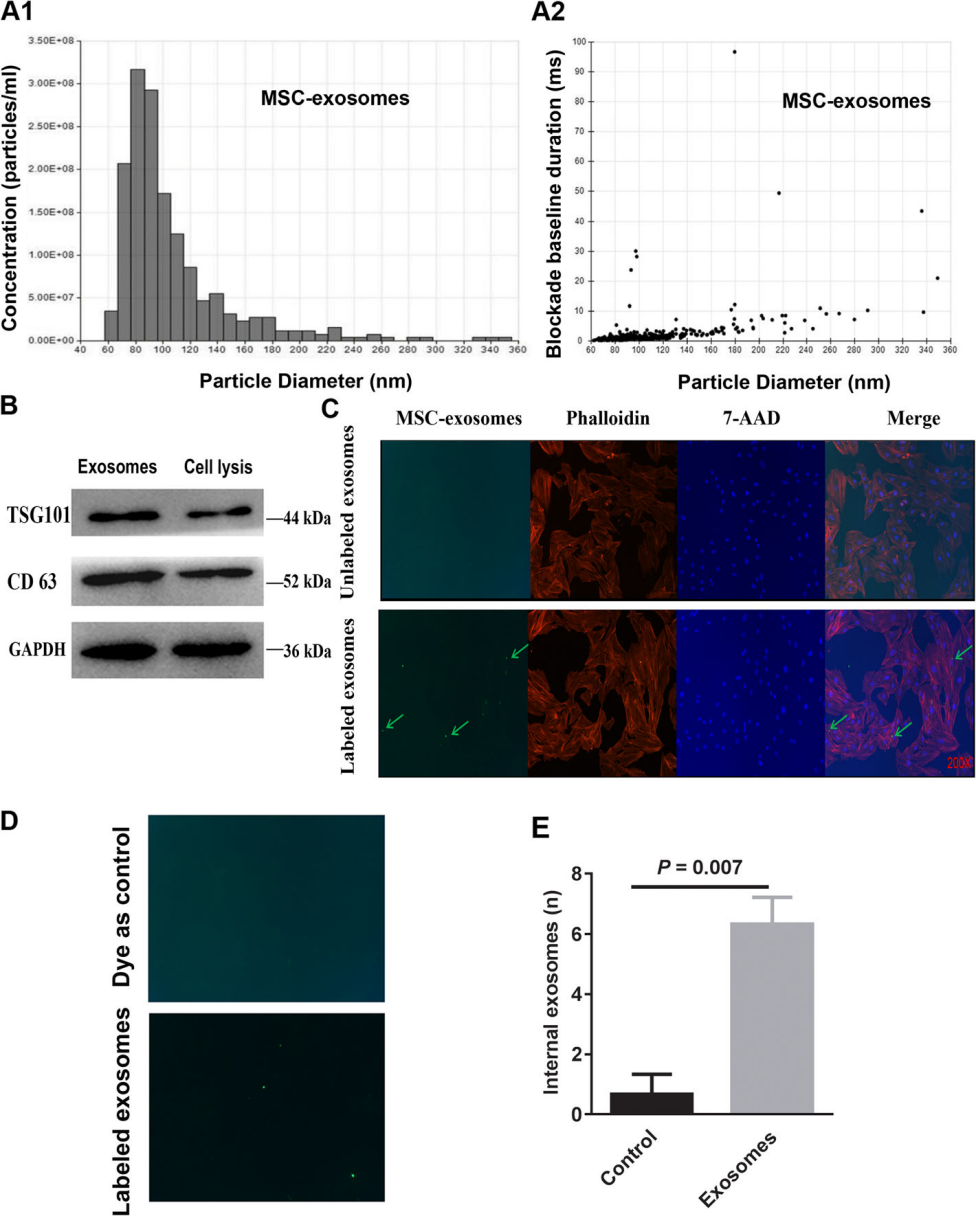
Mesenchymal stem cell (MSC)-derived exosomes were identified and internalized by H9C2 cells. **A1**, **A2** Analysis of the concentration and diameters of MSC-derived exosomes using nanoparticle trafficking. **B** Western blot analysis of the exosome surface markers TSG101 and CD63 (MSC lysis was used as control). **C** H9C2 cells internalized labeled MSC-derived exosomes after a 12-h co-incubation; unlabeled exosomes were used as control. **D–E** Labeled exosomes but not dye alone were readily taken up by H9C2 cells. Statistics calculated based on the results of three repetitions of each experiment

To obtain MSC-derived exosomes, approximately 300 ml of MSC culture media was collected and precipitated. Nanoparticle tracking analysis was used to measure the concentration and size of the exosome particles. The measured concentration of exosome particles was 7.726 × 10^8^ particles/ml and the final concentration of exosomes was determined to be 4.056 × 10^11^ particles/ml; the diameters of the exosome particles were between 80 to 100 nm (Fig. 1A1, A2). Protein quantitation revealed that the concentration of a PBS-exosome solution was 2.551 μg/μl; thus, 1 μg of exosomes included 1.59 × 10^8^ exosome particles. The expression levels of the exosome markers TSG101 and CD 63 were measured using western blotting. Both markers could be detected in MSC-derived exosomes (Fig. 1B). Exosomes labeled with specific fluorescent particles were detected within H9C2 cells, indicating that MSC-derived exosomes could enter the recipient H9C2 CMCs (Fig. 1C). Furthermore, labeled exosomes, but not dye controls, were significantly taken by H9C2 cells (Fig. 1D, E). These findings demonstrated that MSC-derived exosomes could be successfully collected, identified, and internalized into recipient H9C2 cells when co-cultured.

### Establishment of hypoxic cell growth conditions and expression of apoptosis-related protein

**Fig. 2:**
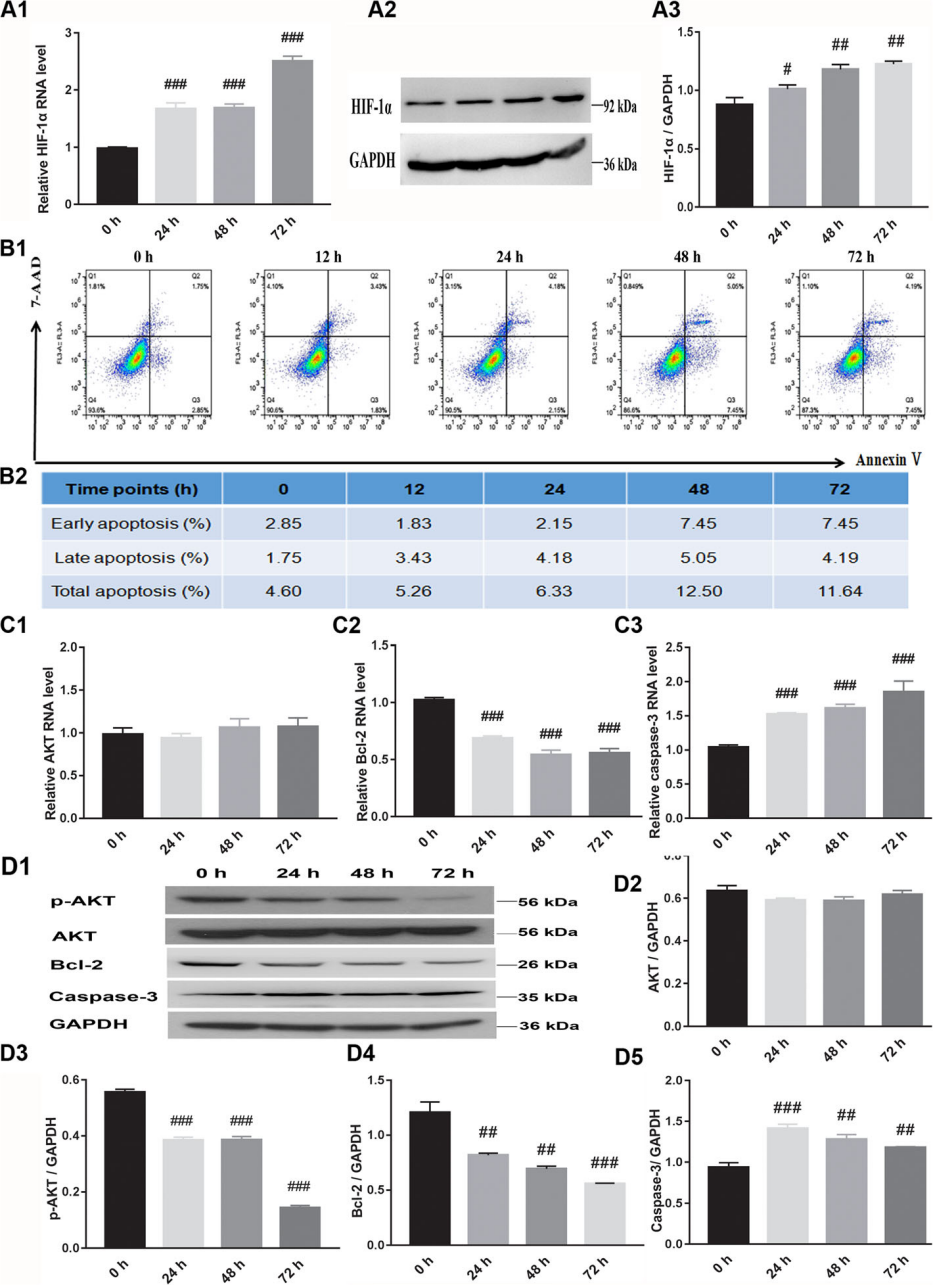
Hypoxia induced apoptosis and altered expression of apoptosis-related protein in H9C2 cells cultured for 0, 12, 24, 48, or 72 h under hypoxic conditions. **A1**–**A3** The expression of HIF-1α gene and protein was determined by real-time PCR and western blotting, respectively. **B1**, **B2** The level of apoptosis was determined by flow cytometry. **C1**–**C3** Expression levels of AKT, Bcl-2, and caspase-3 genes were determined by real-time PCR. **D1**–**D5** Expression levels of AKT, p-AKT, Bcl-2, and caspase-3 were determined by western blotting. ^#^*P* < 0.05, ^##^*P* < 0.01, and ^###^*P* < 0.001; 0-h timepoint was used as control. HIF-1α, hypoxia inducible factor-1α; p-AKT/AKT, phosphorylated protein kinase B; Bcl-2, B-cell lymphoma-2. Statistics calculated based on the results of three repetitions of each experiment

To test the effect of hypoxia on cell apoptosis, the AnaeroPack™ MicroAero system was used to generate hypoxic conditions for cell growth. The expression of both the HIF-1α gene and protein confirmed that the system was effective to create a hypoxic cell growth condition for CMCs (Fig. 2A1–A3). Compared to cells grown in normoxia, H9C2 cells incubated in hypoxic conditions suffered increased apoptotic injury. The number of apoptotic cells peaked after 48 h was selected as the time point for analysis in subsequent experiments (Fig. 2B1, B2). The expression levels of a variety of proteins associated with cell apoptosis, such as AKT/p-AKT, Bcl-2, and caspase-3 were evaluated in our experiments (Fig. 2C1–D5). Our findings revealed that induction of hypoxic conditions gradually decreased the expression of p-AKT, while there was no significant change in the expression of both the total AKT gene and protein. When cells were kept in hypoxic conditions, there were significant differences in both the gene and protein expression levels of Bcl-2 and caspase-3 at each time point tested, when compared to cells grown in normoxia. These findings confirm that the use of the AnaeroPack™ MicroAero system was a reliable approach for establishing a hypoxic cell growth condition for studying cell apoptosis. Changes in the expression of p-AKT/AKT are further explored in later experiments to determine whether the AKT/p-AKT pathway is involved in regulating cell apoptosis in hypoxic conditions created by this system.

### Exosomes protect H9C2 cells from apoptosis and alter expression of anti-apoptotic proteins in hypoxic conditions

**Fig. 3:**
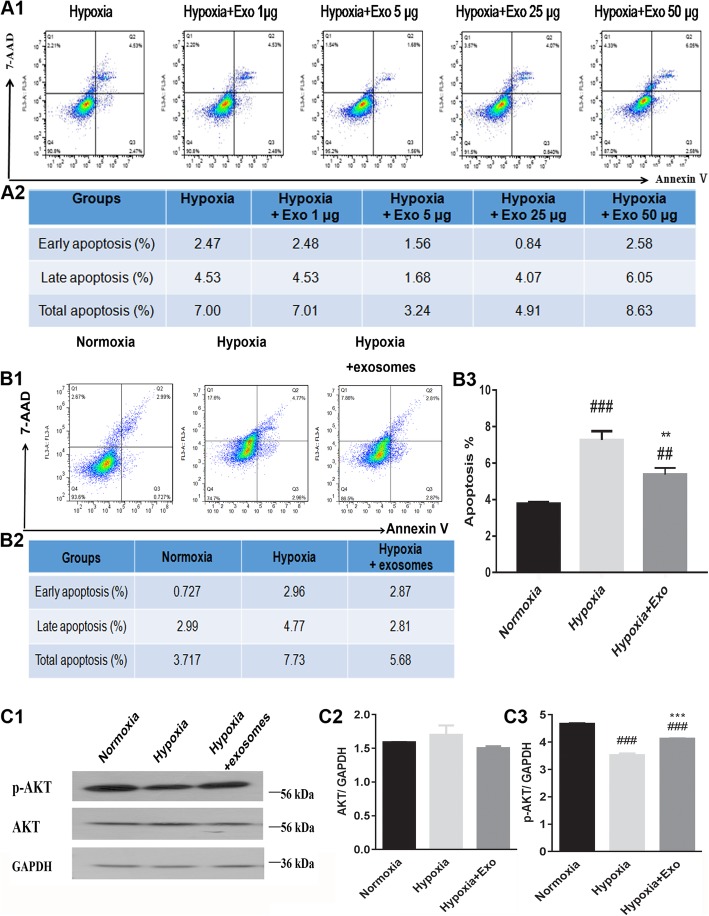
Mesenchymal stem cell-derived exosomes affected H9C2 cell apoptosis and p-AKT/AKT expression levels when cultured in hypoxic conditions. **A1**, **A2** Increasing concentrations of exosomes had an effect on the amount of H9C2 cell apoptosis in hypoxic growth conditions. **B1**–**B3** Flow cytometry analysis showed that exosomes protected H9C2 cells from apoptosis in hypoxic growth conditions. **C1**–**C3** Exosomes regulated the expression levels of p-AKT and AKT in hypoxic growth conditions. ^###^*P* < 0.001 vs. normoxia; ^##^*P* < 0.01 vs. normoxia; ^***^P < 0.001 vs. hypoxia; ^**^P < 0.01 vs. hypoxia. Exo, exosomes; p-AKT/AKT, phosphorylated protein kinase B. Statistics calculated based on the results of three repetitions of each experiment

The present study demonstrated that exosomes released by MSCs were able to enhance the viability of CMCs grown in hypoxic conditions in vitro. Annexin V/7-ADD flow cytometry analysis demonstrated that the amount of apoptosis was markedly decreased compared to control after incubating the cells with exosomes at concentrations of either 5 μg/ml or 25 μg/ml of exosomes prior to induction of hypoxia. However, exosomes added at a low dose (1 μg/ml) did not exert a protective effect against apoptosis in hypoxic conditions, and interestingly, the highest dose of exosomes tested (50 μg/ml) had a pro-apoptotic effect on cells with hypoxic exposure (Fig. 3A1, A2). The increase in apoptosis induced by hypoxic conditions could be attenuated by treating cells with a 5 μg/ml dose of exosomes (Fig. 3B1–B3). Compared to the normoxia control, cells incubated in hypoxic conditions had decreased levels of p-AKT; treating hypoxic cells with MSC-based exosomes was sufficient to restore expression of p-AKT to a greater extent. No significant changes in AKT expression were observed in conditions of normoxia, hypoxia, or exosome treatment in hypoxic conditions (Fig. 3C1–C3). Therefore, MSC-derived exosomes protect H9C2 cells from apoptosis in hypoxic conditions in a dose-dependent manner. These studies indicate that p-AKT/AKT might be an important regulatory pathway in cell apoptosis in the context of hypoxia.

### The role of miR-144 in regulating cell apoptosis and expression of anti-apoptotic proteins

**Fig. 4:**
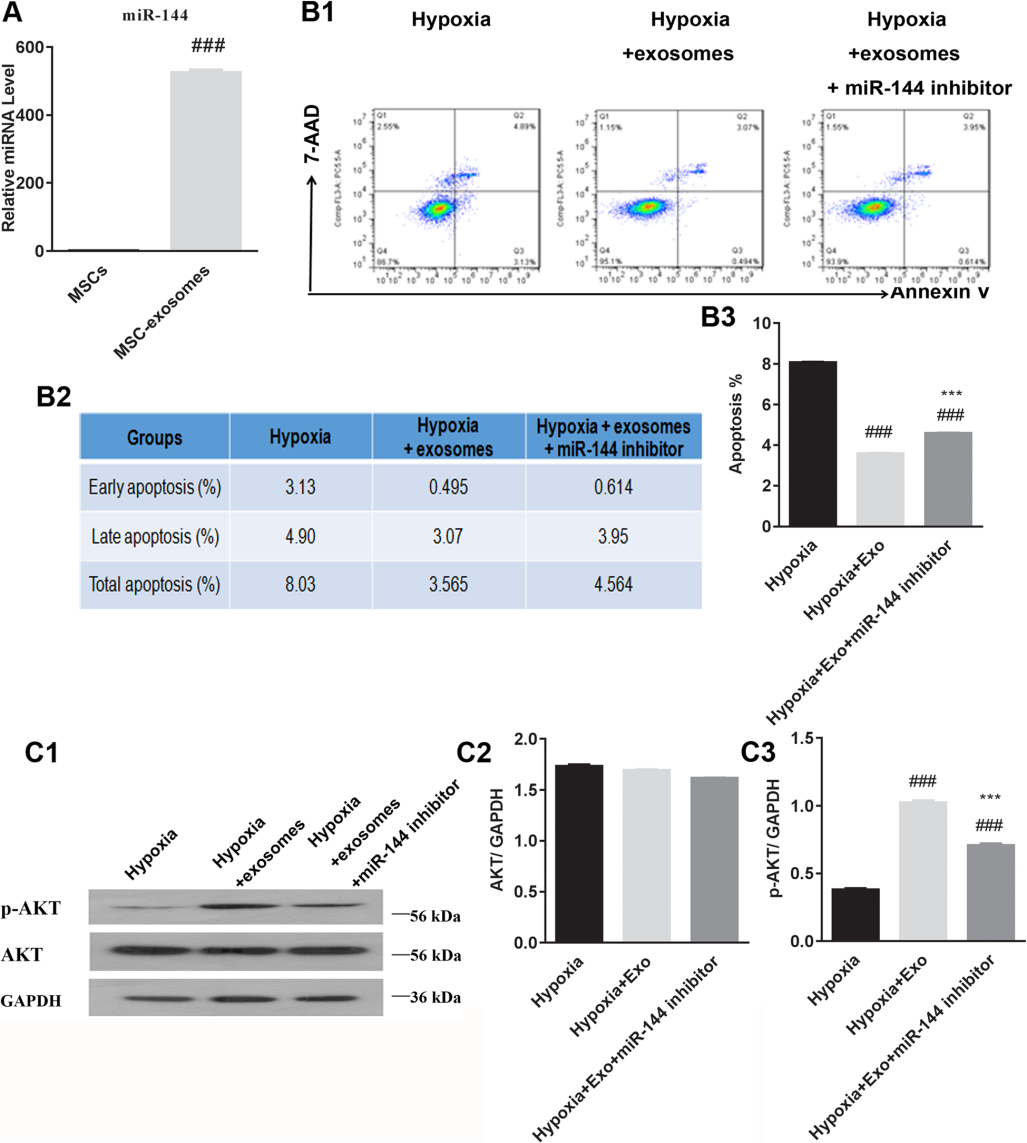
MiR-144 modulates the effect of MSC-derived exosomes on H9C2 cell apoptosis and p-AKT/AKT expression levels. **A** The expression of miR-144 was significantly higher in MSC-derived exosomes than in MSCs. ^###^*P* < 0.001 vs. MSCs. **B1**–**B3** Transfection of H9C2 cells with a miR-144 inhibitor partially inhibited the protective effect of MSC-derived exosomes on cell apoptosis in the context of hypoxia. ^###^*P* < 0.001 vs. hypoxia; ^***^P < 0.001 vs. hypoxia+Exo. **C1**–**C3** Transfection of H9C2 cells with a miR-144 inhibitor modulated the effect of MSC-derived exosomes on changes in p-AKT/AKT expression levels in hypoxic conditions. ^###^*P* < 0.001 vs. hypoxia; ^***^P < 0.001 vs. hypoxia+Exo. MSCs, mesenchymal stem cells; Exo, exosomes; p-AKT/AKT, phosphorylated protein kinase B. Statistics calculated based on the results of three repetitions of each experiment

We found that miR-144 is expressed at a significantly higher level in MSC-derived exosomes than in MSCs themselves, suggesting that MSCs package the majority of the miR-144 produced in exosomes (Fig. 4A). H9C2 cells transfected with a miR-144 inhibitor showed diminished protection from apoptosis in hypoxic conditions when exosomes were pre-incubated with the cells (Fig. 4B1–B3). Transfection of H9C2 cells with the miR-144 inhibitor also attenuated the induction of p-AKT observed with exosome pre-incubation followed by incubation in hypoxic conditions (Fig. 4C1–C3).

**Fig. 5:**
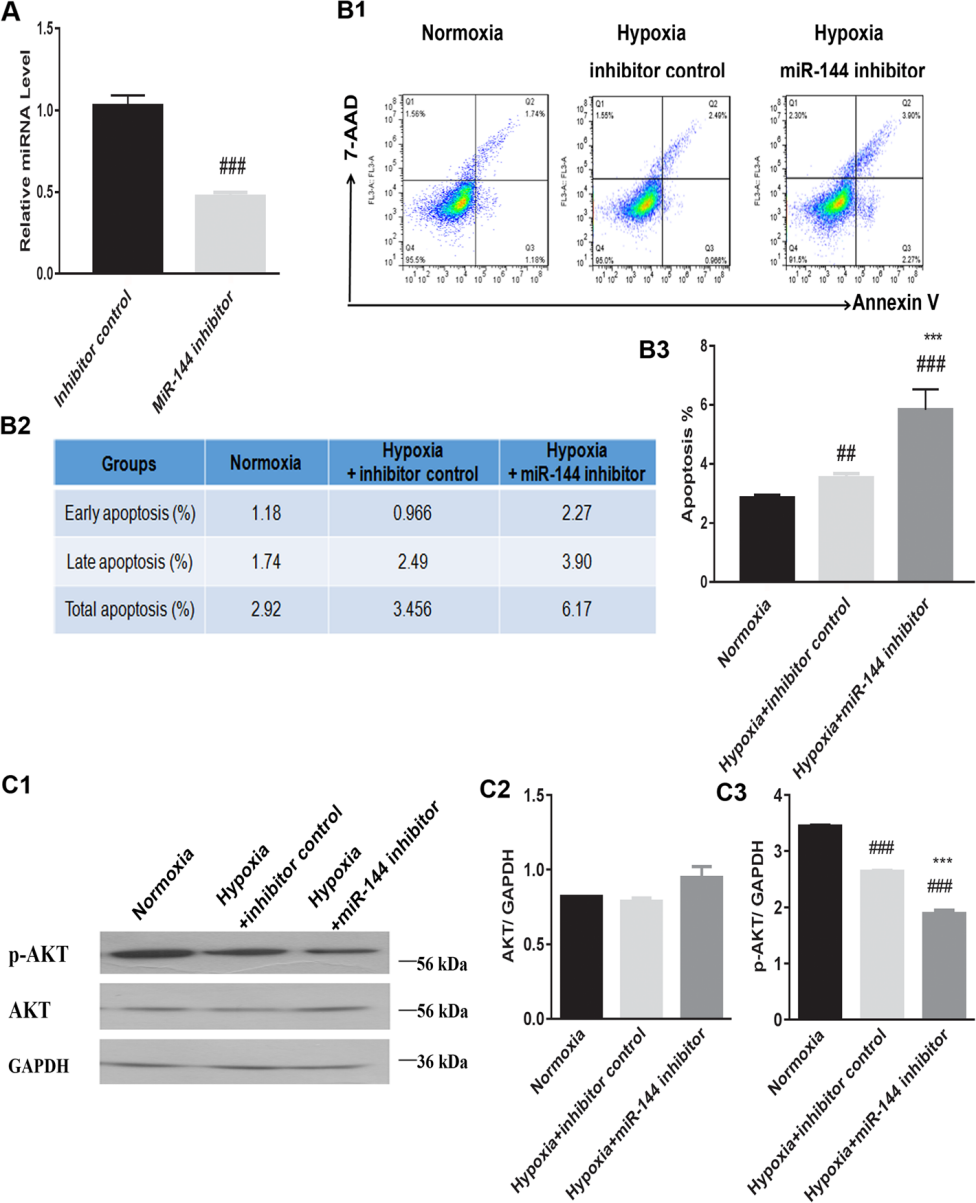
A transfected miR-144 inhibitor alters H9C2 cell apoptosis and changes p-AKT/AKT expression levels in hypoxic conditions. **A** Validation of transfected miR-144 inhibitor along with the inhibitor control by real-time PCR. ^###^*P* < 0.001 vs. inhibitor control. **B1**–**B3** H9C2 cells transfected with the miR-144 inhibitor had increased apoptosis in the induction of hypoxia. ^###^*P* < 0.001 vs. normoxia; ^##^*P* < 0.01 vs. normoxia; ^***^P < 0.001 vs. hypoxia+ inhibitor control. **C1**–**C3** H9C2 cells transfected with the miR-144 inhibitor had altered p-AKT/AKT expression levels in hypoxic conditions. ^###^*P* < 0.001 vs. normoxia; ^***^P < 0.001 vs. hypoxia+ inhibitor control. P-AKT/AKT, phosphorylated protein kinase B. Statistics calculated based on the results of three repetitions of each experiment

**Fig. 6:**
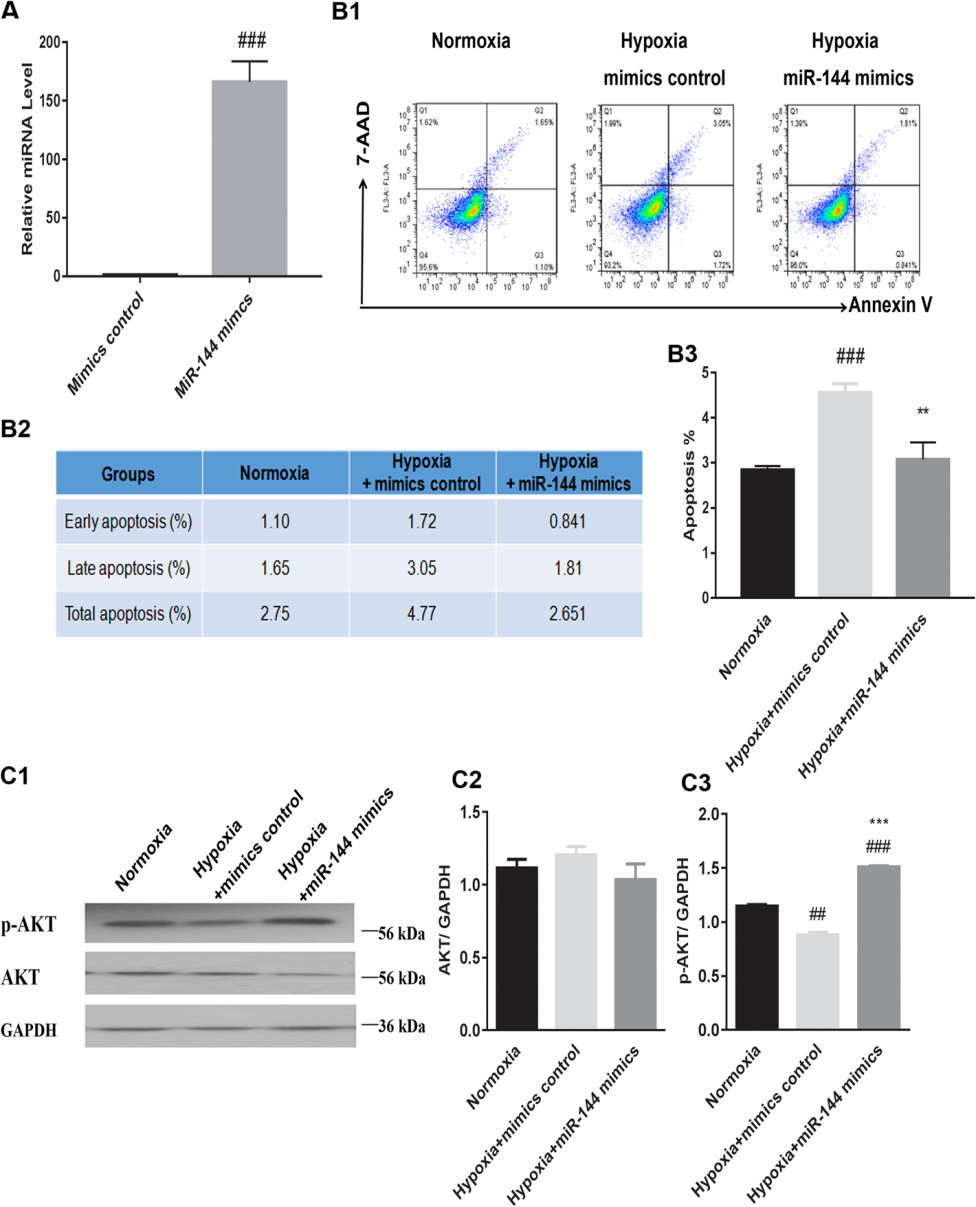
The transfected miR-144 mimics affect H9C2 cell apoptosis and p-AKT/AKT expression levels in the context of hypoxia. **A** Identification of transfected miR-144 mimics and mimics control. ^###^*P* < 0.001 vs. mimics control. **B1**–**B3** H9C2 cells transfected with miR-144 mimics had decreased cell apoptosis in hypoxic conditions. ^###^*P* < 0.001 vs. normoxia; ^**^P < 0.01 vs. hypoxia+ mimics control. **C1**–**C3** H9C2 cells transfected with miR-144 mimics had altered p-AKT/AKT expression levels in hypoxic conditions. ^###^*P* < 0.001 vs. normoxia; ^##^*P* < 0.01 vs. normoxia; ^***^P < 0.001 vs. hypoxia+ mimics control. P-AKT/AKT, phosphorylated protein kinase B. Statistics calculated based on the results of three repetitions of each experiment

Mimics and the inhibitor of miR-144 were transfected into H9C2 cells to explore whether miR-144 alone was sufficient to have an anti-apoptotic effect on hypoxic cells. Transfection with the miR-144 inhibitor exerted a pro-apoptotic effect on H9C2 cells in hypoxic conditions (Fig. 5B1–B3), while miR-144 mimics protected cells from apoptosis in hypoxic conditions (Fig. 6B1–B3). Furthermore, these results indicated that transfection with miR-144 mimics resulted in increased expression of p-AKT in the context of hypoxia (Fig. 6C1–C3), whereas transfection with the inhibitor resulted in decreased expression of p-AKT (Fig. 5C1–C3). However, cells transfected with either the miR-144 mimics or miR-144 inhibitor did not have altered AKT expression patterns in hypoxic conditions. Thus, treatment of hypoxic H9C2s with miR-144 alone recapitulated the anti-apoptotic phenotype observed with addition of MSC-derived exosomes to H9C2s, as well as the pattern of increased p-AKT expression in the hypoxic conditions.

### Potential mechanism for MSC-derived exosomal miR-144 mediated protection of cells from apoptosis in the context of hypoxia

**Fig. 7:**
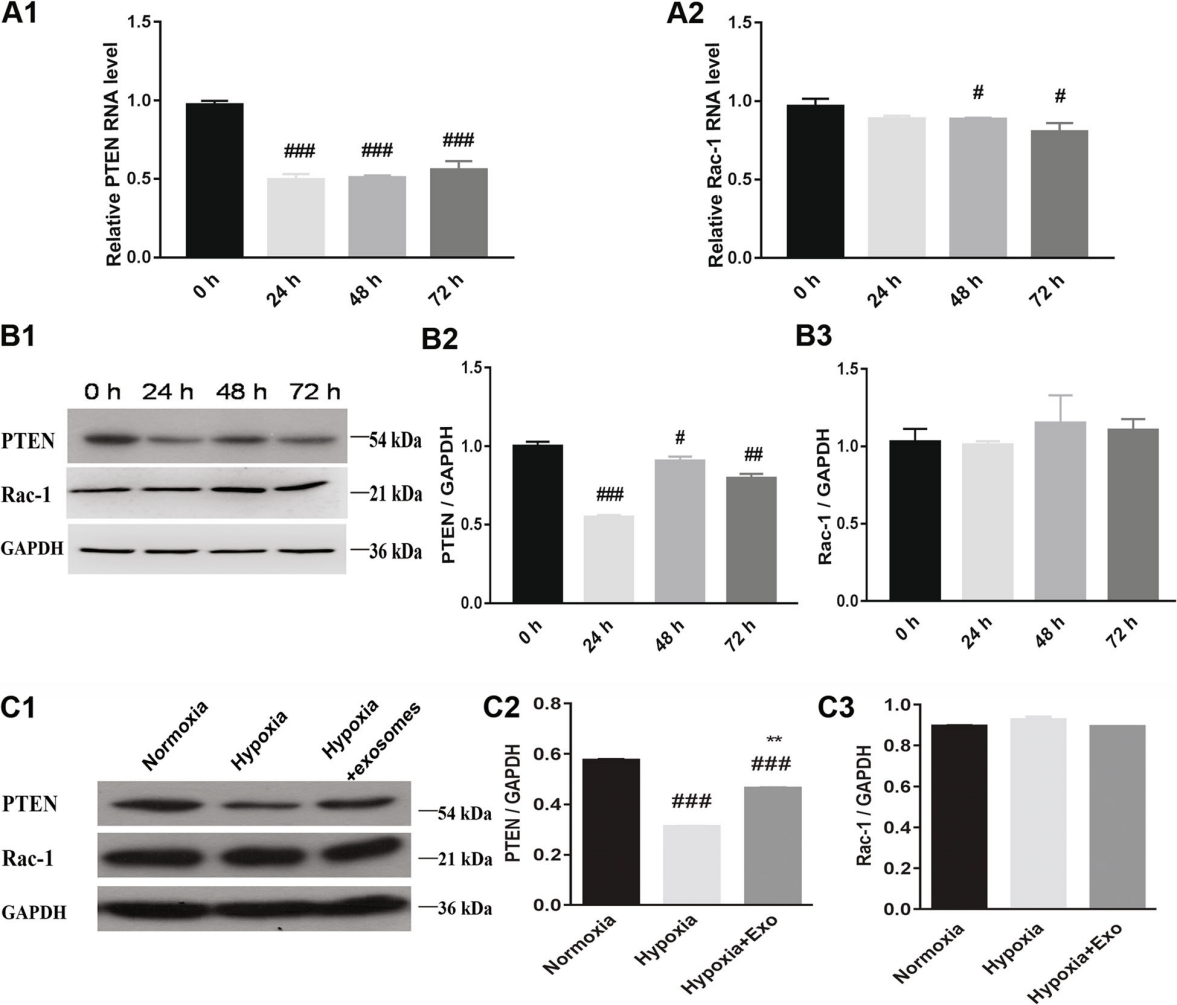
Expression levels of PTEN and Rac-1 in hypoxic conditions and modulated by exosomes. **A1**, **A2** Expression levels of PTEN and Rac-1 after growth in hypoxic conditions for 0, 24, 48, or 72 h, determined from by real-time PCR. ^###^*P* < 0.001, ^#^*P* < 0.05; 0-h timepoint was used as control. **B1**–**B3** Expression levels of PTEN and Rac-1 after growth in hypoxic conditions for 0, 24, 48, or 72 h, determined by western blotting. ^###^*P* < 0.001, ^##^*P* < 0.01, ^#^*P* < 0.05; 0-h timepoint was used as control. **C1**–**C3** Exosomes regulated expression of PTEN and Rac-1 in the context of hypoxia. ^###^*P* < 0.001 vs. normoxia; ^**^P < 0.01 vs. hypoxia. Exo, exosomes; PTEN, phosphatase and tensin homolog deleted on chromosome 10; Rac-1, Ras-related C3 botulinum toxin substrate 1. Statistics calculated based on the results of three repetitions of each experiment

**Fig. 8:**
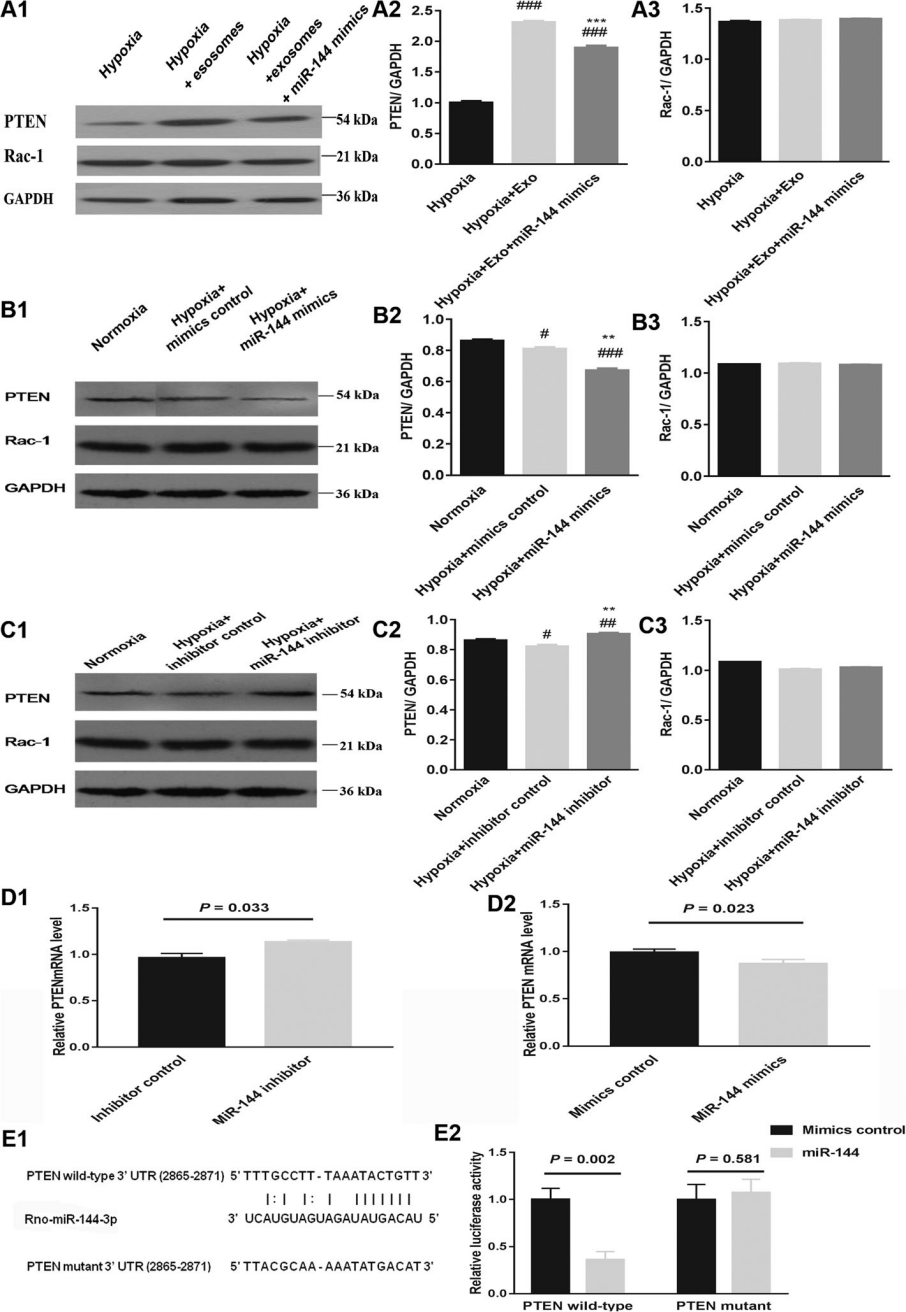
Western blot and real-time PCR analysis of expression levels of potential targets of miR-144. **A1–A3** Transfection of H9C2 cells with miR-144 mimics altered the effect of exosomes on expression of PTEN and Rac-1 in the context of hypoxia. ^###^*P* < 0.001 vs. hypoxia; ^***^P < 0.001 vs. hypoxia+Exo. **B1**–**B3** Transfection of H9C2 cells with miR-144 mimics altered expression levels of PTEN and Rac-1 in hypoxic conditions. ^###^*P* < 0.001 vs. normoxia; ^#^*P* = 0.018 vs. normoxia; ^**^P < 0.01 vs. hypoxia+ mimics control. **C1**–**C3** Transfection of H9C2 cells with the miR-144 inhibitor altered expression levels of PTEN and Rac-1 in the induction hypoxia. ^#^*P* = 0.038 vs. normoxia; ^##^*P* < 0.01 vs. normoxia; ^**^P < 0.01 vs. hypoxia+ inhibitor control. **D1** Transfection of H9C2 cells with the miR-144 inhibitor had increased expression level of PTEN mRNA in hypoxic conditions. **D2** Transfection of H9C2 cells with miR-144 mimics had decreased expression level of PTEN mRNA in hypoxic conditions. **E1** The putative target sequence of PTEN 3′ UTR for miR-144-3p was from the TargetScan prediction software. **E2** Activities of the luciferase in wild-type or mutant PTEN 3′ UTR reporter plasmids were detected by luciferase reporter assay after the transfection of cells with miR-144-3p or control. Exo, exosomes; PTEN, phosphatase and tensin homolog deleted on chromosome 10; Rac-1, Ras-related C3 botulinum toxin substrate 1, UTR, untranslated region. Statistics calculated based on the results of three repetitions of each experiment

Since PTEN and Rac-1 are potential targets of miR-144 and known regulators of the p-AKT/AKT signaling pathway, they were included for analysis in this study. We found that expression of both PTEN and Rac-1 gene were altered in hypoxic conditions, while the protein expression of PTEN but not Rac-1 was altered in hypoxic conditions (Fig. 7A1–B3). Compared to the normoxia control cells, the hypoxic cells had a decreased level of PTEN expression, but pre-incubating the cells with exosomes partially restored PTEN expression in the hypoxia treatment condition (Fig. 7C1–C3). However, the exosome-mediated increase in PTEN expression levels was attenuated in cells transfected with miR-144 mimics (Fig. 8A1–A3). Mimics or an inhibitor of miR-144 were transfected into H9C2 cells to determine their effect on PTEN and Rac-1 expression; transfection with miR-144 mimics decreased the expression of PTEN, whereas cells transfected with the inhibitor had increased PTEN expression (Fig. 8B1–C3). However, there was no significant change in the expression of Rac-1 in the hypoxia treatment condition or with exosome treatment, compared to the normoxia control (Fig. 7B1–C3). Exosome-mediated Rac-1 expression level was not altered in cells transfected with miR-144 mimics, and there was no significant change in the expression of Rac-1 with miR-144 mimics/inhibitor treatment when compared to the hypoxic condition alone (Fig. 8A1–C3). We have also measured the mRNA expression level of PTEN in the context of miR-144 mimics or inhibitor treatment and found that miR-144 inhibitor increased the PTEN mRNA level while miR-144 mimics decreased the PTEN mRNA level (Fig. 8D1, D2). In order to provide more compelling evidence whether miR-144 directly targets the 3′ UTR of PTEN, a luciferase reporter assay was performed to further validate that PTEN is a target of miR-144 (Fig. 8E1, E2). The luciferase activity was significantly inhibited in cells co-transfected with miR-144 and the wild-type PTEN 3′UTR, whereas no effect was observed in cells co-transfected with miR-144 and the mutant PTEN 3′UTR.These findings further confirm that miR-144 accounts for the anti-apoptotic function of MSC-derived exosomes in the context of hypoxia, at least in part by targeting PTEN expression, but not Rac-1.

**Fig. 9:**
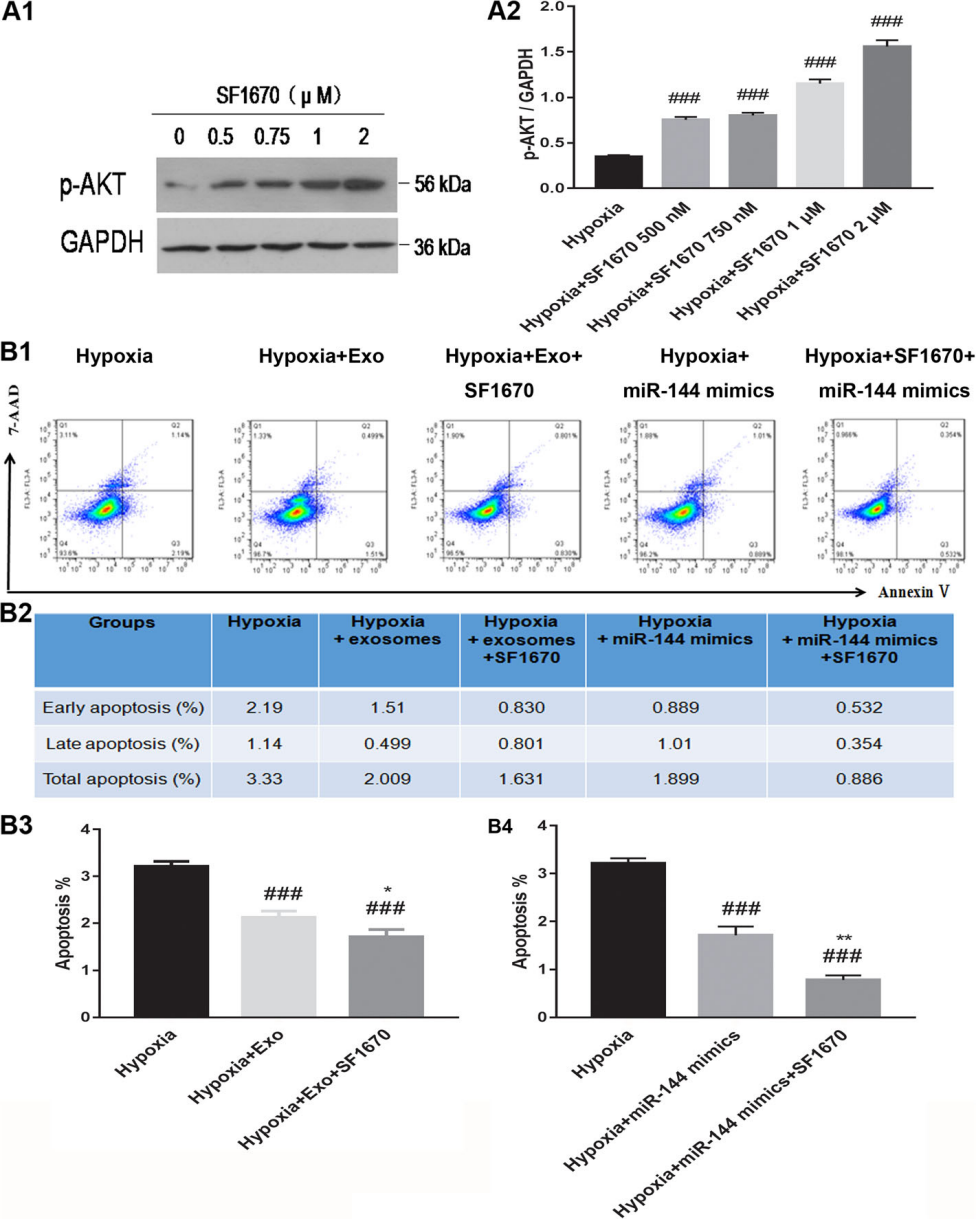
PTEN-specific inhibitor SF1670 increased p-AKT expression levels and modulated H9C2 cell apoptosis in the context of hypoxia. **A1**, **A2** Western blot analysis of p-AKT expression levels under hypoxic growth conditions with addition of varied concentrations of SF1670. **B1**–**B4** SF1670 treatment further decreased H9C2 cell apoptosis compared to exosomes or miR-144 mimic treatment in hypoxic conditions. ^###^*P* < 0.001 vs. hypoxia; ^**^P < 0.01 vs. hypoxia+miR-144 mimics; ^*^P < 0.05 vs. hypoxia+Exo. PTEN, phosphatase and tensin homolog deleted on chromosome 10; p-AKT, phosphorylated protein kinase B; Exo, exosomes. Statistics calculated based on the results of three repetitions of each experiment

SF1670, a PTEN-specific inhibitor, was used to elucidate the role of PTEN in regulating cell apoptosis in hypoxic conditions (Fig. 9). Our results revealed that addition of SF1670 to hypoxic H9C2 cells decreased the expression of p-AKT in a dose-dependent manner (Fig. 9A1, A2). When hypoxic H9C2 cells were co-treated with MSC-derived exosomes and SF1670, the number of apoptotic cells was significantly decreased relative to addition of only MSC-derived exosomes. This suggests that the inhibition of PTEN in hypoxic conditions promotes cell survival. Furthermore, addition of SF1670 in combination with miR-144 mimics to hypoxic H9C2s promoted cell survival to a greater extent than miR-144 mimics alone; this finding indicates that MSC-derived exosomes exert their anti-apoptotic effect at least in part through miR-144 targeting of PTEN (Fig. 9B1–B4). Taken together, these findings show that treating hypoxic H9C2 cells with miR-144 delivered to cells in MSC-derived exosomes promotes cell viability through targeting the PTEN/p-AKT signaling pathway.

## Discussion

Paracrine effects seem to be the predominant mechanism by which MSCs promote CMC viability in hypoxic conditions. Exosomes derived from MSCs have promising potential to be utilized as a novel therapy for ischemic heart disease that might overcome the risks and obstacles typically associated with traditional stem cell therapies. Several studies have demonstrated that MSC-derived exosomes exert cardioprotective effects in vitro and are able to help preserve cardiac function and geometry by reducing infarct size and improving cardiac remodeling after myocardial ischemic injury [27–30]. Consistent with other findings [31–33], exosomes obtained using the reliable exosome isolation kits and confirmed by both NTA and western blotting were efficiently taken up by H9C2 cells in our study. We also found that exosomes released by MSCs enhanced the viability of hypoxic CMCs. However, the appropriate therapeutic concentration of exosomes bears careful consideration, as we found that exosomes exert pro-survival effects in a concentration-dependent manner.

MSC-derived exosomes have been reported to play an important anti-apoptotic role in CMCs both in vivo and in vitro [5–7, 10]. The therapeutic effects of exosomes are mainly mediated by transferring growth factors and various miRNAs to recipient cardiac cells. Our previous findings [3, 4] and other studies [11] demonstrate that highly expressed miRNAs may at least partially account for the protective effects of MSC-derived exosomes on cells in the context of hypoxia. Gain- and loss-of-function studies have revealed that there are signature expression patterns of miRNAs that correlate with repair mechanisms involved in MSC-based therapies in the ischemic myocardium [3, 4, 11]. A growing body of evidence has demonstrated that increasing miR-144 levels in the ischemic myocardium, either by intravenous administration or overexpression, could have cardioprotective effects with improved ventricular function and remodeling [14–16]. In contrast, knockdown of endogenous miR-144 resulted in loss of viability in the same conditions, suggesting miR-144 is required for cardioprotection from ischemic stimulation [15, 16, 34]. However, little is known regarding the relationship between miR-144 and the effects of MSC-derived exosomes on cells in hypoxic growth conditions. We observed that miR-144 was highly abundant in MSC-derived exosomes in the present study. Consistent with other findings that miR-144 has anti-apoptotic effects on CMCs in hypoxic conditions both in vitro and in vivo [17, 35], we have demonstrated that miR-144 contained in MSC-derived exosomes protects CMCs from hypoxic injury by reducing cellapoptosis. MiR-144 mimics and a miR-144 inhibitor were used to further understand the anti-apoptotic functions of miR-144 in the present study. Based on our findings in this study and evidence from other work, miR-144 has promising potential as a therapy for the treatment of ischemic heart disease.

Our findings have revealed that apoptosis is an adverse modulator involved in cardiac repair post-infarction, and several miRNAs exert anti-apoptotic effects by targeting PTEN [2–4, 26]. MiRNAs contained in MSC-derived exosomes could functionally inhibit PTEN expression, thereby activating PI3K/AKT signaling with subsequent inhibition of apoptotic injury and death in hypoxic CMCs [36, 37]. To date, many studies [18–20, 38] have confirmed PTEN as a miR-144 target. Bioinformatics analysis using TargetScan and miRTarBase has also confirmed that PTEN is a good candidate miR-144 target. PTEN is a phosphatase that negatively regulates the PI3K/AKT pathway. Several studies have confirmed that miR-144 promotes cell proliferation and viability through targeting the PTEN/AKT pathway [18, 38]. We found in this study that although miR-144 was highly abundant in MSC-derived exosomes, the expression of PTEN increased in hypoxic H9C2 cells pre-treated with exosomes. This suggests that exosomes containing other miRNAs or growth factors that may enhance the expression of PTEN could diminish the anti-apoptotic effect of MSC-derived exosomes. Additionally, miR-144 mimics induced a marked decrease in the expression of PTEN, resulting in elevated levels of p-AKT, while transfection of miR-144 inhibitor increased the expression of PTEN, which in turn diminished p-AKT levels. Furthermore, we performed a luciferase reporter assay to further validate whether miR-144 directly targeted the 3′ UTR of PTEN and found PTEN as a direct target of miR-144. The pharmacological inhibition of PTEN can further protect H9C2 cells from apoptosis in the context of hypoxia when used in combination with MSC-derived exosomes or miR-144 mimics. Other studies have also indicated that miRNAs can protect against hypoxia-induced cell apoptosis by inhibiting PTEN and targeting the AKT signaling pathway [39, 40]. These findings further support the conclusion that MSC-derived exosomes exert anti-apoptotic effects on hypoxic CMCs by delivering miR-144 to cells where it targets the PTEN/AKT pathway (Additional file 2: Figure S1). Notably, the expression of Rac-1 was not changed in hypoxic condition and was not altered by MSC-derived exosomes as well as miR-144 mimics or the miR-144 inhibitor.

## Conclusions

In summary, MSC-derived exosomes exert an anti-apoptotic effect on cells in hypoxic conditions, at least in part through miR-144-mediated regulation of the PTEN/AKT signaling pathway. Exosomes from MSCs have cardioprotective function, independent of stem cell differentiation or stemness, making MSC-derived exosomes a promising potential to be used in cell-free cardiotherapy for ischemic heart disease. The activity of the miR-144/PTEN/AKT pathway in hypoxic conditions was tested at only one time point after treatment with MSC-derived exosomes, which is an important limitation of the present study. Second, findings from the hypoxic model in our current study cannot be simply extrapolated to other hypoxic conditions, and human cardiac CMCs isolated from the ventricles of the adult heart but not H9C2 cell line, as well as a bench-top hypoxia workstation and incubator rather than the AnaeroPack™ MicroAero system, should be preferably used for myocardial research in hypoxic conditions in the future. In addition, the proposed underlying mechanisms of this study have not been extensively tested using in vivo models, which is a major limitation of the present study. We are planning in vivo experiments to study the mechanism of miR-144-mediated cardioprotection in the future. While there are several hundred miRNAs known to be expressed in MSCs, and some roles for these miRNAs in MSCs have been elucidated, the specific expression patterns and functionality of many miRNAs in MSC-derived exosomes remain unknown. Furthermore, it is still unclear which exosomal miRNAs are the best modulators for use in developing MSC-based therapies. Addressing these unknowns will help pave the way for utilization of exosomes as a non-invasive stem cell therapeutic vehicle to deliver miRNAs therapies to alleviate ischemic myocardial damage.

## Supplementary information

Additional file 1: **Table S1.** PCR primer sequences.

Additional file 2: **Figure S1.** Potential mechanisms based on conclusions from present study. Abnormal expression of PTEN in H9C2 cells in hypoxic growth conditions leads to decreased expression of p-AKT, resulting in increased cell apoptosis. MicroRNA-144 included in MSC-derived exosomes exerts anti-apoptotic effects by targeting and silencing PTEN to protect cells from hypoxia-induced apoptosis. Alternatively, pharmacologic inhibition of PTEN with SF1670 can also be used to achieve anti-apoptotic effects in hypoxic growth conditions.

## Data Availability

All data generated or analyzed during this study are included in this published article.

## Abbreviations

AKT: Protein kinase B
Bcl-2: B-cell lymphoma-2
BSA: Bovine serum albumin
CMCs: Cardiomyocytes
FBS: Fetal bovine serum
HIF-1α: Hypoxia inducible factor-1α
MSCs: Mesenchymal stem cells
NTA: Nanoparticle trafficking analysis
p-AKT: Phosphorylated protein kinase B
PBS: Phosphate-buffered saline
PI3K: Phosphatidylinositol 3-kinase
PTEN: Phosphatase and tensin homolog deleted on chromosome 10
Rac-1: Ras-related C3 botulinum toxin substrate 1
UTR: Untranslated region
