# Supplementary material for: Mesenchymal stem cell-derived exosomes ameliorate cardiomyocyte apoptosis in hypoxic conditions through microRNA144 by targeting the PTEN/AKT pathway
Source: Stem Cell Res Ther. 2020 Jan 23;11:36. doi: 10.1186/s13287-020-1563-8 (PMC6979357; doi:10.1186/s13287-020-1563-8)
Supplement: Supplementary file 1 — Table S1. PCR primer sequences. [file 13287_2020_1563_MOESM1_ESM.docx]

| Table S1. PCR primer sequences | | |
| --- | --- | --- |
| Genes | Sequence | Length (bp) |
| PTEN |  | 184 |
| Rat-PTEN-F | AGACCATAACCCACCACAGC |  |
| Rat-PTEN-R | CAGGGCCTCTTGTGCCTTTA |  |
| Rac-1 |  | 99 |
| Rat-Rac-1-F | GAACGGCTCGGATAGCTTCA |  |
| Rat-Rac-1-R | CCATGGCGAAAGAGATCGGT |  |
| AKT-1 |  | 273 |
| Rat-AKT-1-F | AGGCATCCCTTCCTTACAGC |  |
| Rat-AKT-1-R | CAGCCCGAAGTCCGTTATCT |  |
| Bcl-2 |  | 194 |
| Rat-Bcl-2-F | ACTCTTCAGGGATGGGGTGA |  |
| Rat-Bcl-2-R | TGACATCTCCCTGTTGACGC |  |
| Caspase-3 |  | 147 |
| Rat-caspase-3-F | AGCTGGACTGCGGTATTGAGA |  |
| Rat-caspase-3-R | CATGACCCGTCCCTTGAATT |  |
| HIF-1α |  | 195 |
| Rat-HIF-1α-F | GTTTCTGCTGCCTTGTAT |  |
| Rat-HIF-1α-R | ACTATGTCGCTTTCTTGG |  |
| GAPDH |  | 74 |
| Rat-GAPDH-F | GCAAGAGAGAGGCCCTCAG |  |
| Rat-GAPDH-R | TGTGAGGGAGATGCTCAGTG |  |
| U6 |  | 96 |
| Rat-U6-F | CTCGCTTCGGCAGCACA |  |
| Rat-U6-R | AACGCTTCACGAATTTGCGT |  |
