# Supplementary figures and images for: Mesenchymal stem cell-derived exosomes ameliorate cardiomyocyte apoptosis in hypoxic conditions through microRNA144 by targeting the PTEN/AKT pathway
Source: Stem Cell Res Ther. 2020 Jan 23;11:36. doi: 10.1186/s13287-020-1563-8 (PMC6979357; doi:10.1186/s13287-020-1563-8)

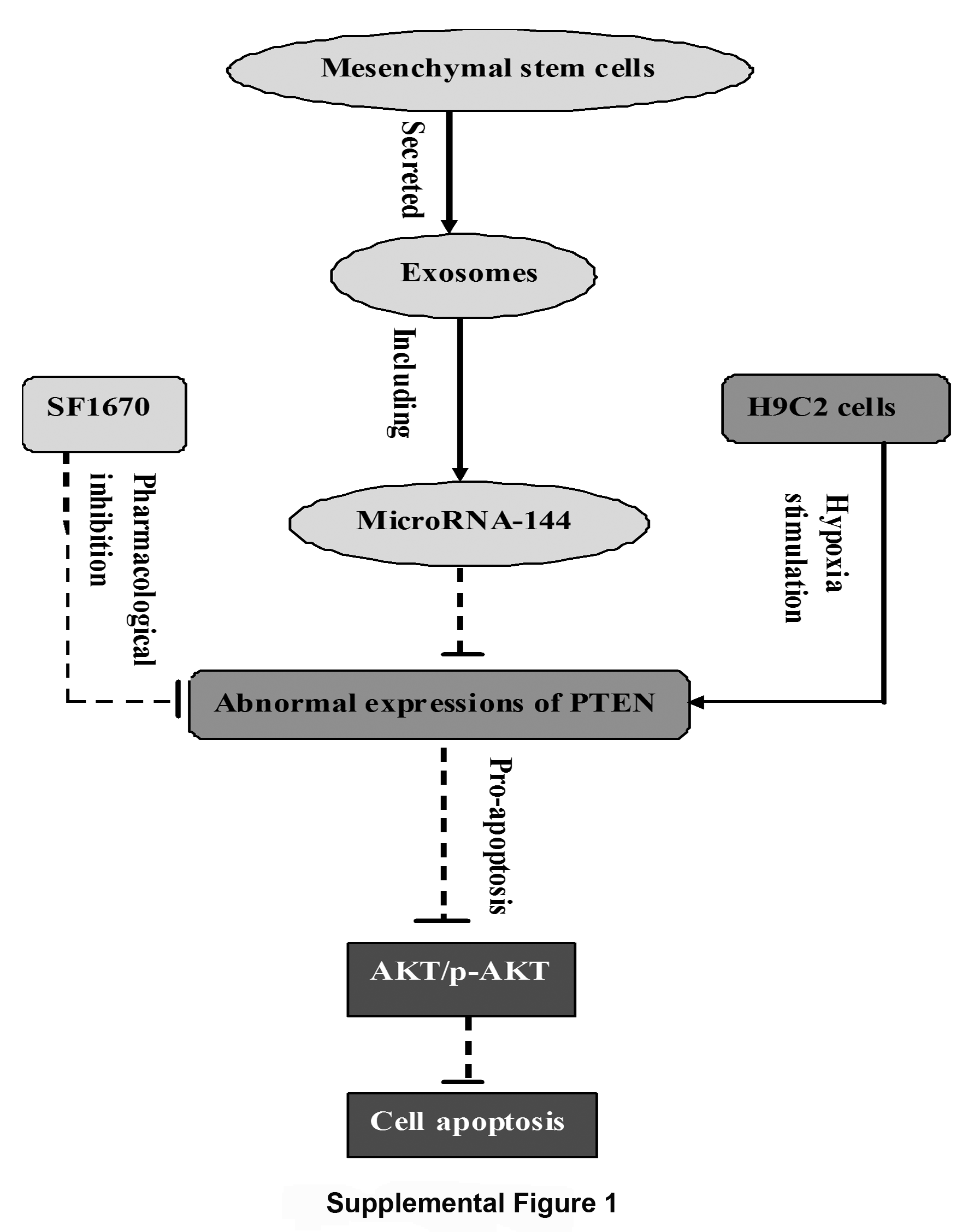

Supplement: Supplementary file 2 — Figure S1. Potential mechanisms based on conclusions from present study. Abnormal expression of PTEN in H9C2 cells in hypoxic growth conditions leads to decreased expression of p-AKT, resulting in increased cell apoptosis. MicroRNA-144 included in MSC-derived exosomes exerts anti-apoptotic effects by targeting and silencing PTEN to protect cells from hypoxia-induced apoptosis. Alternatively, pharmacologic inhibition of PTEN with SF1670 can also be used to achieve anti-apoptotic effects in hypoxic growth conditions. [file 13287_2020_1563_MOESM2_ESM.tif]
